# Novel Mechanisms in Heart Failure With Preserved, Midrange, and Reduced Ejection Fraction

**DOI:** 10.3389/fphys.2019.00874

**Published:** 2019-07-05

**Authors:** Ishan Lakhani, Keith Sai Kit Leung, Gary Tse, Alex Pui Wai Lee

**Affiliations:** ^1^Department of Medicine and Therapeutics, Faculty of Medicine, Chinese University of Hong Kong, Hong Kong, China; ^2^Faculty of Medicine, Li Ka Shing Institute of Health Sciences, Chinese University of Hong Kong, Hong Kong, China; ^3^Aston Medical School, Aston University, Birmingham, United Kingdom

**Keywords:** inflammation, phenomapping, LV dyssynchrony, network analysis, mortality

## Introduction

Heart failure (HF) represents a major epidemic with high morbidity and mortality rates, imposing a significant burden on healthcare systems worldwide (Savarese and Lund, 2017). HF has long been distinguished by ejection fraction (EF) into two types—HF with reduced ejection fraction (HFrEF), for which EF is below 40%, and HF with preserved ejection fraction (HFpEF), for which EF is above 50% and, according to the 2016 European Society of Cardiology (ESC) Guidelines (Ponikowski et al., 2016), accompanies (1) an elevated level of natriuretic peptides (BNP > 35 pg/ml and/or NT-proBNP > 125 pg/mL) and (2) the presence of either structural heart disease (left ventricular hypertrophy and/or left atrial enlargement) or diastolic dysfunction. HFrEF and HFpEF were initially considered to be binary opposing entities at two ends of the same spectrum. However, whilst several studies have demonstrated the efficacy of drug therapies in improving quality-of-life and long-term clinical outcomes in HFrEF patients, such pharmacological approaches have often failed to yield similar observable benefits in HFpEF cohorts. As such, the current paradigm follows that the pathogenesis underscoring the development and progression of HFrEF and HFpEF are distinct. In more recent developments, the 2016 ESC Guidelines (Ponikowski et al., 2016) also proposed a third class of HF–HF with mid-range ejection fraction (HFmrEF), for which EF is between 40 and 49%, and accompanies the same two aforementioned components of HFpEF. Investigations into this newly defined group of HF patients have yielded contradicting results: whilst some findings have demonstrated an overlap between HFmrEF and the other two classes, others have shown no such association. As a result, a greater understanding of the underlying mechanistic differences between the HF groups, particularly pertaining to HFpEF and HFmrEF, is still needed in order to ensure successful diagnoses and holistic treatment provision.

**Figure 1:**
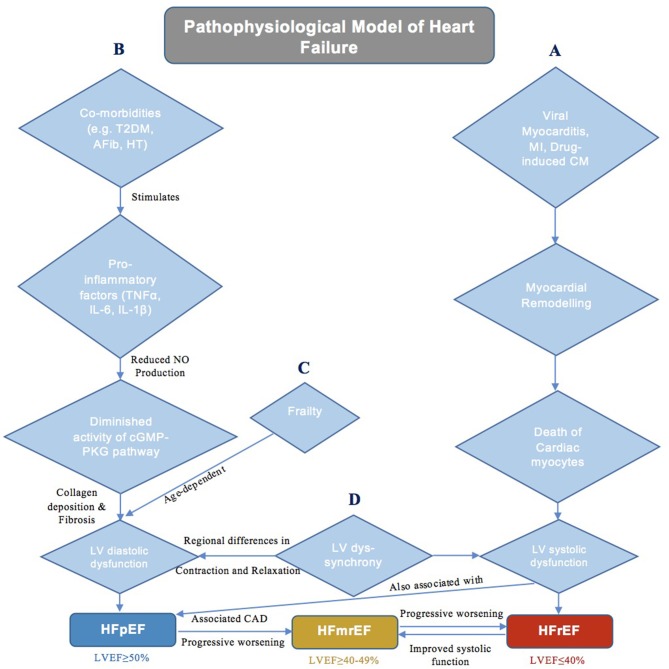
A schematic diagram demonstrating the current theories of underlying pathophysiology in different classes of heart failure. **(A)** Pathophysiology of HFrEF. **(B)** Pathophysiology of HFpEF. **(C)** Role of Frailty in HFpEF. **(D)** Role of LV Dyssynchrony in HF.

The proposed mechanism for HFrEF is generally well-understood, in which adverse myocardial remodeling, resulting from cardiomyocyte death (Gonzalez et al., 2011) secondary to an inciting stimulus, such as viral myocarditis, myocardial infarction, or drug-induced cardiomyopathy (Bloom et al., 2017), leads to systolic dysfunction (Figure 1A). The same however cannot be said for HFpEF, which is instead associated with a more heterogeneous pathophysiology (Kao et al., 2015). Epidemiological studies have illustrated a comparatively stronger relationship between HFpEF (as opposed to HFrEF) with multiple cardiac and non-cardiac co-morbidities, including but not limited to type 2 diabetes mellitus (T2DM), arterial hypertension, renal failure, obesity, and atrial fibrillation (Elguindy and Yacoub, 2012). This evidently diverse clinical phenotype has elicited much debate regarding the precise mechanisms involved in the development of HFpEF.

## Systemic Proinflammatory Hypothesis

One potential hypothesis suggests that HFpEF is simply the additive outcome of the many associated co-morbidities acting synergistically (Kao et al., 2015). Paulus et al. proposes a mechanism that lends credence to this notion by indicating that the concurrent existence of conditions such as T2DM, obesity, arterial hypertension, and pulmonary disease is responsible for inducing a systemic proinflammatory state (Figure 1B), characterized by elevated levels of tumor necrosis factor (TNF)-α, interleukin (IL)-6, and IL-1ß, amongst many others (Van Linthout and Tschöpe, 2017). Such cytokines in turn initiate a series of signaling events that ultimately culminate in reduced endothelial nitric oxide (NO) production and diminished activity of the cyclic guanosine phosphate-protein kinase G (cGMP-PKG) pathway in cardiomyocytes. This cascade of reactions eventually results in cardiomyocyte stiffness coupled with myocardial collagen deposition and fibrosis, therein leading to the development of hypertrophy, diastolic dysfunction and HFpEF (Paulus and Tschöpe, 2013). This theory has been supported not only by various animal models demonstrating the protective role of NO-cGMP-PKG signaling against myocardial hypertrophy (Calderone et al., 1998) and stiffness (Matsubara et al., 1998), but also by certain investigations showing the efficacy of anti-inflammatory agents (statins) in reducing mortality in HFpEF patients (Liu et al., 2014; Alehagen et al., 2015; Marume et al., 2019). Moreover, it must be noted that the aforementioned systemic proinflammatory state is, in fact, also involved in the pathogenesis of HFrEF, whereby in addition to cardiomyocyte death secondary to an inciting stimulus, elevated levels of IL-6 and TNF-α also mediate a reduction in NO-cGMP-PKG signaling that contributes to myocardial dysfunction (Paulus and Tschöpe, 2013). However, despite the apparent importance of inflammation in the pathogenesis of HFpEF (and HFrEF), a significant proportion of conducted clinical trials have also demonstrated the ineffectiveness of anti-inflammatory statins as well as vasoprotective ACE inhibitors (Fu et al., 2012) and angiotensin II receptor antagonists (Yusuf et al., 2003) in HFpEF cohorts, thereby somewhat diminishing the credibility of the systemic proinflammatory hypothesis.

## Multi-Organ Disease Hypothesis

An alternative theory is the belief that HFpEF, rather than being a single disease, instead results from the interaction of multiple underlying physiological ailments. These include not only a reduction in diastolic function and cardiac reserves but also impairments in the renal and pulmonary systems (Borlaug, 2014), all of which collectively show significant inter-individual variations (Roh et al., 2017). The severity of these conditions is age-dependent (Parikh et al., 2018), and frailty (Tse et al., 2018; Zhang et al., 2018), a syndrome resulting from an age-related reduction in physiological function, itself has been linked with adverse outcomes in HF (Figure 1C). All in all, this notion of a differential phenotypic expression resulting from a complex interplay of multiple comorbidities is well-accepted, and likely accounts for the failure of conventional pharmacological therapies used in HFrEF to yield the same beneficial outcomes in HFpEF. Nonetheless, recent efforts have been made to construct animal models that closely mimic HFpEF phenotype. Obesity as well as salt-driven hypertensive models have both been used to accurately study respiratory muscle weakness and associated exercise intolerance in HFpEF. Seiler et al. describes salt-loaded hypertensive HFpEF rats with diaphragmatic muscle alterations secondary to elevated plasma levels of inflammatory cytokines (TNF-α, IL-1β, etc.), consistent with the systemic proinflammatory hypothesis (Seiler et al., 2016). Moreover, models of renal insufficiency-driven hypertension have also shown to present with LV hypertrophy and poor LV relaxation, reflective of the diastolic dysfunction related to HFpEF (Munagala et al., 2005). However, as previously stated, HFpEF is characterized by multiple comorbidities that interact to produce to the final phenotype. This cannot be replicated by animal models, which normally elucidate the mechanistic role of only one particular comorbidity (e.g., arterial hypertension, obesity, renal insufficiency, etc.) in the development of HFpEF. Whilst this approach allows for an understanding of the relationship between each individual comorbidity and HFpEF (Valero-Muñoz et al., 2017), it will likely serve to benefit only a subset of patients for whom the investigated comorbidity is the predominant factor contributing to disease pathogenesis.

## LV Dyssynchrony

Both the systemic proinflammatory state and multi-organ disease hypotheses encompass the role of left ventricular diastolic dysfunction (LVDD) in HFpEF development. LVDD has long been considered the major causative factor of HFpEF; however, many previous trials aiming to reduce long-term mortality by enhancing diastolic function, namely by improving LV relaxation and/or halting the progression of LV hypertrophy through the antagonism of the renin-angiotensin-aldosterone system, have failed to report favorable outcomes (Cleland et al., 2006; Massie et al., 2008). Such findings have warranted and fueled the search for other contributing mechanisms central to the pathogenesis of HFpEF for prospective targeting in the clinical setting. One such alternative that is becoming increasingly investigated is LV dyssynchrony (Figure 1D), which stems from the regional variations in the rate of contraction and relaxation of fibers in the myocardium, in turn resulting in impaired cardiac performance (Lee et al., 2011). Although it is said to exist in ~30–40% of HFrEF patients (Liu et al., 2018), LV dyssynchrony and its severity have also now been associated with the development of HFpEF (Lee et al., 2011; Santos et al., 2014). Lee et al. showcased not only the existence of systolic and diastolic dyssynchrony at rest in an HFpEF cohort relative to normal controls but also the subsequent aggravation of dyssynchrony when HFpEF patients were exposed to dobutamine-induced hemodynamic stress (Lee et al., 2010). Moreover, Morris et al. demonstrated that the development of LV dyssynchrony is, in fact, associated with subendocardial fibrosis that occurs secondary to a vast majority of comorbidities typically accompanying the HFpEF phenotype (Morris et al., 2012). Such dyssynchrony has been implicated in the development of systolic dysfunction in HFpEF patients, in which delayed myocardial activation diminishes pump efficiency (Cheng et al., 2009). In addition, it has also been postulated that asynchronous LV contraction leads to temporal variability in LV relaxation, particularly during early diastole, wherein certain myocardial fibers relax later than others (Bonow et al., 1988). This subsequently decreases diastolic function, presenting as a reduction in passive LV filling and an increase in LV filling pressures (Morris et al., 2012). All in all, the dynamicity of mechanical dyssynchrony, along with the resulting systolic and diastolic dysfunction, are clearly important to the manifestation of HFpEF, and in turn provide reason as to why therapies that focus solely on the improvement of diastolic function without any regard for LV dyssynchrony have failed to yield fruitful outcomes.

## Mechanisms of HFmrEF

In contrast to HFpEF and HFrEF, there is a relative paucity in literature discussing the phenotype of HFmrEF, and as such, this condition has often been referred to as the “middle child” in the HF family. The pathophysiology of HFmrEF, which accounts for ~10–20% of all HF patients (Nadar and Tariq, 2018), is largely unknown. Current evidence indicates that HFmrEF may be the outcome of a progressive worsening in LV function in HFpEF patients, most notably observed in those with concomitant coronary artery disease, which is associated with a deterioration in LVEF (Lam and Solomon, 2014). An alternative possibility is that HFmrEF results from an improved systolic function in patients with HFrEF. This particular subset of HFmrEF is clinically relevant, as recovered systolic function in HF patients has been linked with reduced mortality and a more favorable long-term prognosis (Nadruz et al., 2016). Regardless of its pathway of development, many studies have presented HFmrEF as an intermediate phenotype with clinical characteristics and outcomes in between the other two classes of HF, albeit more closely related to HFrEF (He et al., 2009; Rickenbacher et al., 2017; Lund et al., 2018). Although such findings of an intermediate clinical profile would support the notion of HFmrEF as a distinct condition—a subset of neither HFpEF nor HFrEF—additional studies are still required for this to be confirmed and for the pathogenesis of HFmrEF to be further elucidated.

## Cardiac Imaging, Biomarkers, and Network Analysis

The heterogeneity of HF has necessitated the use of various techniques in cardiac imaging for diagnosis and prognosis assessment, namely transthoracic echocardiography (TTE), cardiac computerized tomography, and magnetic resonance imaging (Butler, 2007; Inamdar and Inamdar, 2016). Moreover, three-dimensional speckle tracking in echocardiography, a relatively recent development, has also proven to be an accurate, relatively efficient method for assessing LV function in research, thereby warranting its more frequent implementation in the clinical setting for the evaluation of HF (Luo et al., 2014; Xu et al., 2014). However, in addition to the aforementioned cardiac imaging methods, numerous biomarkers related to the pathological processes of HF, such as inflammation (Petersen and Felker, 2006; Bozkurt et al., 2010), myocardial mechanical stress, and fibrosis (Ahmad et al., 2014; López et al., 2015; Li et al., 2018), cardio-renal dysfunction (Senthong et al., 2017), and even the ovarian cancer marker cancer antigen-125 (Cheung et al., 2018), have been identified and investigated. Such biomarker studies perhaps provide some of the most important avenues through which the diversity of HF can be further understood. Recently, network analysis has been implemented as a tool to study different multi-marker interactions for the purpose of obtaining a more comprehensive overview of the distinct pathogenesis of the three classes of HF. Thus, far, this summative, all-encompassing approach has somewhat unsurprisingly revealed multiple shared protein-protein based relationships among HFpEF, HFmrEF, and HFrEF. However, findings have also indicated varying biomarker signatures within the different HF groups. Most notably, HFpEF pathways were uniquely associated with markers of inflammation whilst HFrEF pathways were enriched with markers of cardiac stretch and cellular proliferation, both showing minimal overlapping with HFmrEF (Tromp et al., 2017, 2018). The identification of such specific biochemical interactions not only emphasizes the unique pathophysiology of the three types of HF but also provides insight into potential drug targets, and in turn, suggests the need for dynamic multi-marker screening for optimal risk stratification.

## Phenomapping

In addition to network analysis, another method that has more recently been implemented to assess the underlying heterogeneity in HF, amongst various other diseases, is “phenomapping,” which involves machine-based learning to analyze large sums of phenotypic data in order to categorize patients into distinct subgroups based on a select number of clinical features (Katz et al., 2017). Katz et al. demonstrated the use of this technique to successfully identify distinct subgroups of hypertensive patients with a predisposition to HFpEF development owing to the presence of abnormal cardiac mechanical properties (Katz et al., 2017). Similarly, Shah et al. also used machine learning to stratify an HFpEF cohort according to a series of unrelated clinical phenotypic features found in the domains of patient demographics, physical characteristics, as well as laboratory, ECG and echocardiography parameters. This classification yielded three distinct “pheno-groups” of HFpEF patients, all of whom presented with variable baseline data. Pheno-group #1 was characterized by low levels of brain natriuretic peptide (BNP), which is typically associated with obesity. A lack of BNP promotes renal sodium and water retention, culminating in an increase in plasma volume, preload, and LV hypertrophy. As a result, such a “BNP deficiency syndrome” could likely serve as a possible explanation for the underlying etiology of diastolic dysfunction and subsequent HFpEF in this particular subset of patients. Pheno-group #2 encompassed a phenotype of cardio-metabolic disease, which can disturb myocardial electrical and mechanical function, whilst pheno-group #3 consisted of patients with the highest BNP levels, but concurrent right ventricular and renal dysfunction that predisposed to the highest risk of hospitalization and mortality (Shah et al., 2015; Shah, 2017). With the findings of these investigations, it is evident that phenomapping can serve as a tool to circumvent the problem of heterogeneity in HF by categorizing patients into distinct, clinically relevant clusters. The identification of such subgroups would subsequently allow for the development of patient-specific, targeted therapies that could potentially improve long-term prognosis.

## Conclusion

The three classes of HF are all characterized by distinct pathophysiological processes, which in turn contribute to the heterogeneity in the expressed phenotype. Whilst there is currently less unknown about HFrEF, literature pertaining to HFpEF and the newly classified HFmrEF still incites many questions with respect to pathogenesis and optimal therapeutic strategies. Specifically, more prospective studies, as opposed to cross-sectional studies, that follow HFpEF and HFmrEF patients for a lengthy time period are required to assess whether or not the development of HF is truly, in fact, a continuum that progresses from HFpEF through to HFmrEF and eventually HFrEF. Overall, the diversity in the observed clinical features, namely of HFpEF and HFmrEF, poses challenges to the creation of a universal treatment regimen that will fit all patients. As such, future management of these two conditions, in particular, will likely necessitate the implementation of a patient-specific method that targets unique metabolic derangements present in distinct patient subgroups. Although tools such as network analysis and machine-based learning that attempt to collate and find links between available data have proven to yield informative results, a greater understanding of the underlying mechanisms involved in the development of HFpEF and HFmrEF is still required before this individualistic approach can be employed holistically in the clinical setting.

## Author Contributions

All authors listed have made a substantial, direct and intellectual contribution to the work, and approved it for publication.

### Conflict of Interest Statement

The authors declare that the research was conducted in the absence of any commercial or financial relationships that could be construed as a potential conflict of interest.
